# Active Living Inputs to Cognition: an Exploration of a Hypothesis

**DOI:** 10.1002/alz70860_096986

**Published:** 2025-12-23

**Authors:** Ezinne Ekediegwu, Nancy Mayo

**Affiliations:** ^1^ McGill University, Montreal, QC, Canada

## Abstract

**Background:**

Active living is a broad concept that includes exercise, recreational activities, household and occupational tasks, and active transportation to combat sedentary behaviour. It addresses social issues related to inactivity, especially among older adults, rather than focusing solely on physical fitness.

As populations age, cognitive health has become a public health priority due to its impact on daily functioning and quality of life. Mayo et al. suggest that active living, influenced by physical and mental capacities and social determinants of health, could promote cognitive health in older adults. Identifying modifiable contributors to cognitive health can help reduce the burden of cognitive decline.

**Objective:**

The study aims to identify active living factors that are associated with self‐reported cognitive ability and to identify profiles of people with differing degrees of self‐reported cognitive ability.

**Methods:**

A secondary analysis of a cross‐sectional study was conducted using survey responses from 1,612 older adults (65+ years) from Canada, the UK, the USA, and the Netherlands. The survey covered self‐reported cognitive ability, personal factors, intrinsic capacity factors, SDOH, and active living indicators.

The outcome was measured using the short form of the Communicating Cognitive Concerns Questionnaire (C3Q), which assesses the frequency of memory and attention lapses. Personal factors included age, sex, gender, and health conditions. Intrinsic capacity factors included sensory impairments, symptoms, and physical capacity. SDOH included nationality, education, spirituality, ethnicity, finance, residence, services, resources, neighborhood agreeableness, and social support. Active living indicators were measured using the Older Persons Active Living Related Quality of Life (OPALrQOL) measure.

**Analysis:**

Multivariable analysis identified active living factors associated with self‐reported cognitive ability. Logistic regression and classification tree analysis were used to identify significant predictors and profiles of cognitive ability.

**Results:**

Significant predictors of cognitive ability included fatigue, anxiety/depression, resilience, health status, motivation, hearing ability, well‐being, pain interference, and social support. Fatigue was the most critical factor, followed by anxiety/depression and resilience.

**Conclusion:**

Factors such as anxiety, fatigue, and lack of resilience negatively impact cognition. However, resilience can mitigate these effects. The study's findings can inform public health policies and interventions to promote cognitive health through active living.

